# ﻿*Hypericumshunhuangshanense* (Hypericaceae), a new species from Hunan, China

**DOI:** 10.3897/phytokeys.260.153988

**Published:** 2025-07-11

**Authors:** Xi-Ya Ou, Zhao-Fu Chu, Wen-Xiang Ouyang, Wei-Qiang Qin, Zhi-Xin Quan, Meng-Hua Zhang, Dai-Gui Zhang, Jia-Wei Xiao

**Affiliations:** 1 College of Agriculture and Forestry Ecology, Shaoyang University, Shaoyang, 422004, Hunan, China; 2 Hunan Shunhuangshan National Nature Reserve Administration, Shaoyang, 422700, Hunan, China; 3 School of Marxism, Jishou University, Zhangjiajie, 427000, Hunan, China; 4 College of Biology and Environmental Sciences, Jishou University, Jishou, 416000, Hunan, China

**Keywords:** ITS sequences, Hunan Shunhuangshan National Nature Reserve, Molecular phylogeny, Morphology

## Abstract

*Hypericumshunhuangshanense*, a new species of Hypericaceae from the Shunhuangshan National Nature Reserve in Hunan Province, China, is described and illustrated, based on morphological and molecular phylogenetic evidence. The new species resembles *H.faberi* in morphology but clusters with *H.seniawinii* in phylogenetic analyses; it can be easily distinguished from both by its leaves sessile and decussate, inflorescence cymose, anthers yellow to deep orange, and locules 3.

## ﻿Introduction

The genus *Hypericum* L. belongs to the family Hypericaceae, with over 500 species globally (Robson 2016), predominantly found in temperate areas of the Northern Hemisphere and tropical high-altitude mountains (Crockett and Robson 2011). In China, there are 68 species and nine subspecies of this genus, which are primarily herbs or shrubs, occasionally trees, featuring flowers that are typically yellow, golden or sometimes white. Robson (1972, 1977, 1985, 1990, 2001, 2006, 2010a, 2010b, 2012, 2016) has conducted detailed monographic and molecular phylogenetic studies on *Hypericum*, classifying the genus into 36 sections. Nürk et al. (2015) used ITS sequences to analyze the phylogenetic relationships within *Hypericum*. Their results showed that one of these sections, H.sect.Ascyreia, was non-monophyletic.

During 2024, a floristic survey was conducted in the Shunhuangshan National Nature Reserve, Hunan, China and an unusual species of Hypericaceae was discovered. This species should be placed in Hypericumsect.Hypericum since its stamen fascicles apparently 3, styles 3, sepals, bracts, and bracteoles margin entire. However, it is easily distinguished from other species in this section by its sessile and decussate leaves, and cymose inflorescence. After careful morphological comparison and molecular phylogenetic analyses, we confirmed this population represents a previously undescribed species, formally described here as *Hypericumshunhuangshanense*.

## ﻿Material and methods

**Figure 1.**
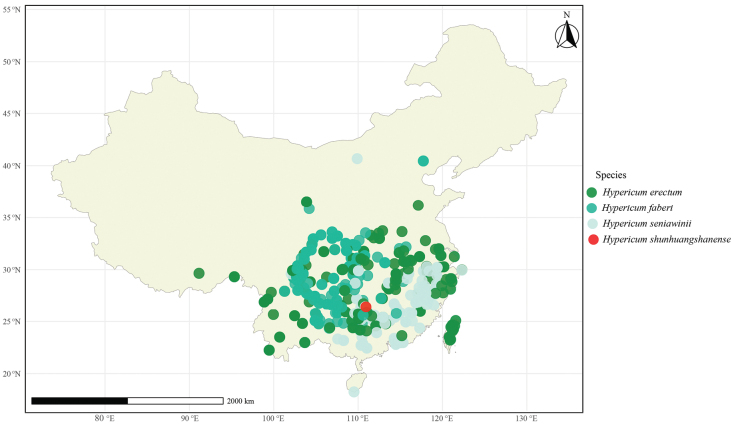
Distribution of *Hypericumshunhuangshanense*, *H.erectum*, *H.faberi* and *H.seniawinii*.

**Figure 2.**
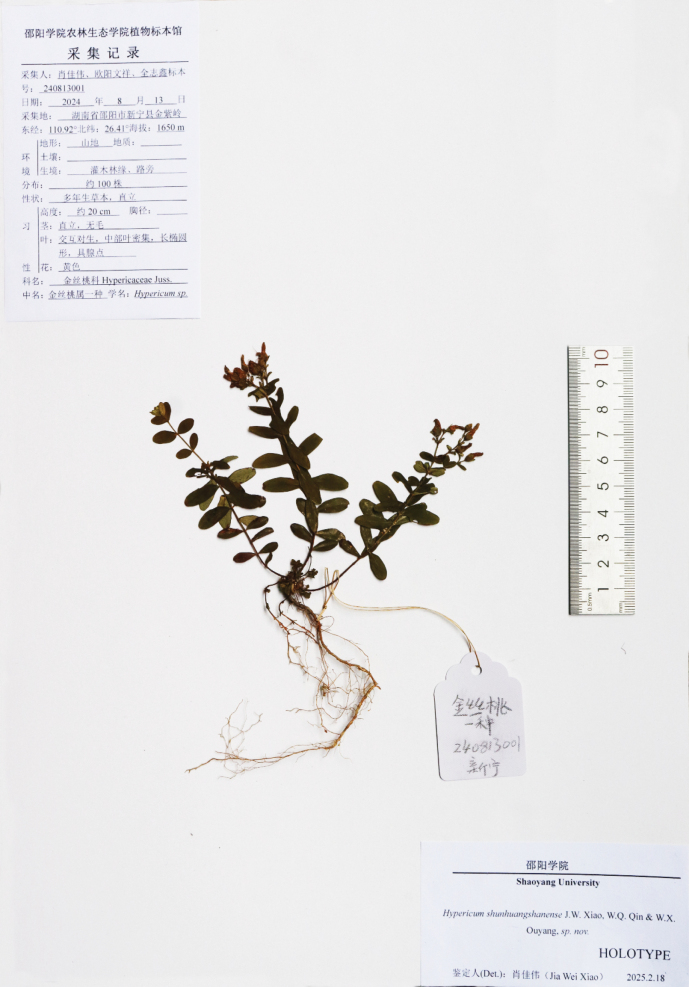
Holotype of *Hypericumshunhuangshanense* J.W. Xiao, W.Q. Qin & W.X. Ouyang.

Specimens of the new taxon were collected in Xinning County (Fig. 1), Hunan, China. We collected leaf material and dried it with silica gel for molecular experiments. The voucher specimens have been deposited in the herbarium of Shaoyang University (Fig. 2) and Jishou University (240813001, 240813002).

The description of the new species is based on living material, dry specimens and FAA fixed materials. Eighteen individuals were examined. The morphological comparison with similar species, such as *H.erectum*, was based on the study of herbarium specimens (http://plants.jstor.org/) and Chinese Virtual Herbarium (https://www.cvh.ac.cn/). We compared plant height, stem characteristics, presence or absence of petioles, leaf arrangement, sepal size, inflorescence, anther color, distribution of glandular dots, petal length, and number of locules in the ovary.

The data from ITS sequences of 54 species, representing major clades of the genus *Hypericum* (Nürk et al. 2013; Wu et al. 2024), were downloaded from GenBank (Table 1). Besides, two newly-sequenced accessions of two individuals of *H.shunhuangshanense* from Xinning County were included (Table 1). *Thorneacalcicola* (Standl. & Steyerm.) Breedlove & E.M.McClint. was selected as outgroup following Park and Kim (2004). The total genomic DNA of the two accessions (Table 1) was isolated from silica gel-dried leaves using a modified cetyltrimethylammonium bromide procedure (Doyle and Doyle 1987). Amplification and sequencing were performed using the primers ITS1 and ITS4 (White et al. 1990) for the ITS region. The PCR amplification was carried out according to Meseguer et al. (2014). PCR products were purified using a UNIQ-10 Spin Column PCR Product Purification Kit (Sangon Biotech Co., Ltd., Shanghai, China) following the manufacturer’s instructions. Sequencing reactions were performed in both directions by Sangon Biotech Co., Ltd.

**Table 1.** The information on samples used for phylogenetic inference in this study.

Species	GenBank No.
*H.androsaemum* L.	KC709337
*H.attenuatum* Choisy	HE662752
*H.balearicum* L.	AY555862
*H.barbatum* Jacq.	FJ694192
*H.bupleuroides* Griseb.	HE653429
*H.calycinum* L.	HE653431
*H.canariense* L.	KC709387
*H.concinnum* Benth.	HE653442
*H.elatoides* R. Keller	HE653456
*H.elodeoides* Choisy	HE653457
*H.elodes* L.	FJ694200
*H.empetrifolium* Willd.	HE653464
*H.erectum* Thunb.	JN811119
*H.faberi* R. Keller	MH808697
*H.forrestii* (Chittenden) N. Robson	HE653476
*H.geminiflorum* Hemsl.	HM162838
*H.grandifolium* Choisy	KC709385
*H.heterophyllum* Vent.	HE653492
*H.hirsutum* L.	HE653500
*H.humifusum* L.	HE653507
*H.hypericoides* (L.) Crantz	KC709376
*H.japonicum* Thunb.	HE653513
*H.kalmianum* L.	FJ694209
*H.kamtschaticum* Ledeb.	HE653516
*H.kouytchense* Lévl.	FJ694210
*H.lagarocladum* N. Robson	HE662703
*H.liboense* M.T. An & T.R. Wu	OR981944
*H.monanthemum* Hook. f. & Thomson ex Dyer	HE653542
*H.monogynum* L.	HE653544
*H.nagasawai* Hayata	LK871658
*H.orientale* L.	HE653565
*H.pallens* Banks & Sol.	AY555848
*H.papillare* Boiss. & Heldr.	HE653570
*H.papuanum* Ridl.	HE653571
*H.patulum* Thunb.	FJ694214
*H.perforatum* L.	JN811136
H.perforatumsubsp.veronense (Schrank) H. Lindb.	MN036448
*H.petiolulatum* Hook. f. & Thomson ex Dyer	HE653582
*H.polyphyllum* Boiss. & Balansa	HE662730
*H.prolificum* L.	MT551029
*H.przewalskii* Maxim.	JF976672
*H.pseudohenryi* N. Robson	KC709447
*H.pseudolaeve* N. Robson	HE653594
*H.pseudomaculatum* Bush	HE653595
*H.pseudopetiolatum* R. Keller	AY573002
*H.pulchrum* L.	FJ694219
*H.quartinianum* A.Rich.	HE653603
*H.reflexum* L.f.	HE662747
*H.rumeliacum* Boiss.	HE653616
*H.sampsonii* Hance	HE653620
*H.seniawinii* Maxim.	HE653630
*H.shunhuangshanense* 1 J.W. Xiao, W.Q. Qin & W.X. Ouyang	PV826159
*H.shunhuangshanense* 2 J.W. Xiao, W.Q. Qin & W.X. Ouyang	PV815611
*H.vacciniifolium* Hayek & Siehe	HE653656
*H.wilsonii* N. Robson	HE653658
*H.xylosteifolium* N. Robson	HE653659
*H.yezoense* Maxim.	AY573004
*Thorneacalcicola* (Standl. & Steyerm.)Breedlove & E.M.McClint.	AY573028

DNA sequences were aligned initially using Clustal X1.83 (Jeanmougin et al. 1998), performed by MUSCLE v.3.8.31 (Edgar 2004) and adjusted manually in PhyDE v.0.9971 (Müller et al. 2010). The optimal model of DNA substitutions was selected using the Akaike Information Criterion (Akaike 1973) as applied in jModelTest 2.1.4 (Darriba et al. 2012) prior to the Maximum Likelihood (ML) analyses and Bayesian Inference (BI). GTR+I+G was recommended as the best fit model for ITS. The phylogenetic trees were constructed using Maximum Likelihood (ML) and Bayesian Inference (BI). ML and BI analyses were conducted using RAxML 7.2.6 and MrBayes 3.1.2 (Huelsenbeck and Ronquist 2001; Stamatakis 2006), respectively. For BI, four chains, each starting with a random tree, were run for 1,000,000 generations with trees sampled every 1000 generations. The average standard deviation of split frequencies (< 0.01) was used to assess the convergence of the two runs. After the first ca. 25% discarded as burn-in, the remaining trees were imported into PAUP* v.4.0b10 and a 50% majority-rule consensus tree was produced to obtain posterior probabilities (PP) of the clades. Before the datasets were combined, the incongruence length difference test (Farris et al. 1994) was performed on PAUP* v.4.0b10 (Swofford 2001). Character state changes were equally weighted and gaps were treated as missing data. The aligned lengths of ITS is 802 bp.

## ﻿Results

The morphological characteristics of our putative new species, *H.faberi*, *H.seniawinii*, and *H.erectum* are presented in Table 2. The diagnostic characters of the new species include: curved or flattened and swollen stems, absence of petioles, decussate leaves, racemose inflorescences, sepals laminar, glands pellucid, streaks, and trilocular ovaries. *Hypericumfaberi* differs from the new species primarily in its geniculate or prostrate-ascending stems, petioles 1–3 mm long, and unilocular ovaries. *Hypericumseniawinii* is characterized by erect stems, opposite leaves, trichasium inflorescences, and black or or rarely a few pale glandular dots distributed on the leaves, sepals, and petals. *Hypericumerectum* is distinguished from the new species by its erect stems, opposite leaves, corymbose cyme inflorescences, sepals laminar, glands black, streaks and dots, and petals laminar, glands black, streaks. Importantly, the new species is readily distinguished from its three closely related species by its decussate leaf arrangement, sessile leaves, and cymose inflorescence.

**Table 2.** Morphological comparisons of *Hypericumshunhuangshanense* and similar species.

Characters	*H.shunhuangshanense*	*H.faberi*	*H.seniawinii*	*H.erectum*
Plant height	0.1–0.2 m	0.2–0.8 m	(0.15–)0.3–0.6 m	0.3–0.7 m
Stems	curved or prostrate and swollen	rising in a geniculate or prostrate manner	upright	erect
Petiole	sessile	1–3 mm	sessile	sessile
Leaf arrangement	decussate	opposite arrangement	opposite arrangement	opposite arrangement
Leaf glandular dots	marginal glands black, spaced, rather large	laminar glands absent or more rarely few or scattered dots, pale and rarely black, large; intramarginal glands rather dense, black	laminar gland dots pale, dense, rather large; intramarginal glands all black or the occasional one pale, dense	laminar glands black [and sometimes pale], ± numerous, small; intramarginal glands black, dense
Calyx size	3–5 × 1–2 mm	1.5–2 × 0.8 mm	2.5–3.5 × 1–1.5 mm	2.5 × 1 mm
Sepal glandular dots	laminar glands pellucid, streaks; marginal glands black	laminar glands pale or black, streaks to dots; marginal glands black	laminar glands pale, lines to streaks; marginal glands all black or rarely a few pale	laminar glands black, streaks and dots; intramarginal glands black, (rather dense to) sparse or absent
Petal glandular dots	laminar glands pellucid, streaks; marginal glands black, few	laminar glands absent; marginal glands black, few, near apex only	laminar glands pale, streaks to dots, or absent; marginal glands black, distal	laminar glands black, streaks, distal; marginal glands black,
petal length	1.0–1.5 × 0.5–0.7 cm	0.6 × 0.3 cm	0.7–0.8 × 0.25 cm	0.7 × 0.25 cm
Inflorescence	cymose inflorescence	dichasial cyme	trichasium	corymbose cyme
Number of ovary chambers	three	one	three	three

**Figure 3.**
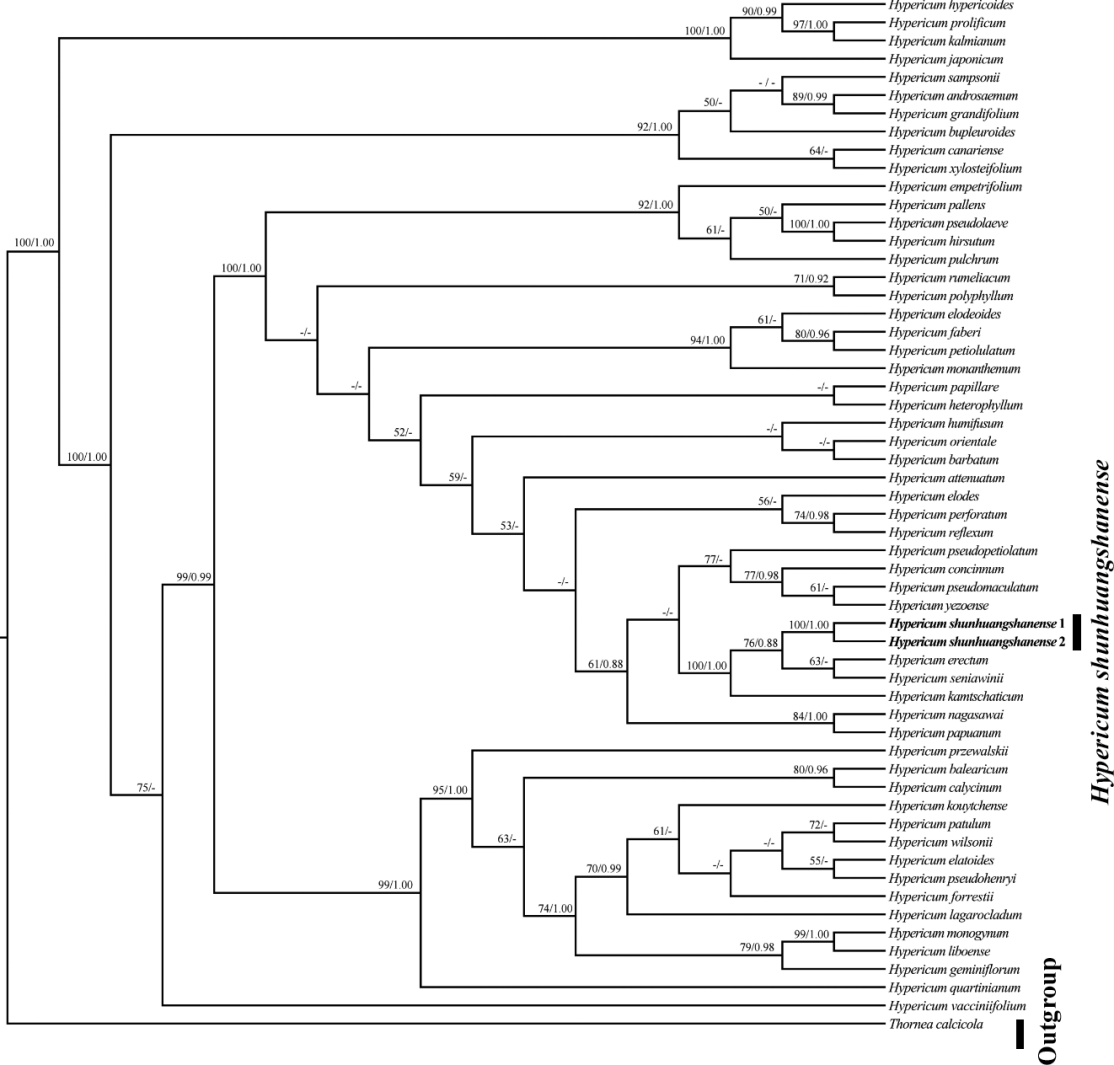
The phylogram of the Maximum Likelihood (ML) tree from the ITS sequences, showing the phylogenetic position of *Hypericumshunhuangshanense*. Bootstrap support values (1000 replicates) for Maximum Likelihood (ML ≥ 50%, left) and Bayesian posterior probabilities (PP ≥ 0.88, right) are provided above the branches. *H.shunhuangshanense* is shown in bold.

ML and BI analyses produced similar topology and only the ML tree is presented in Fig. 3, with ML bootstrap (BS) and PP values for each clade. Phylogenetic analyses strongly supported the monophyly of *H.shunhuangshanense* (PP = 1.00, BS = 100%). This clade showed a sister relationship to a moderately supported clade comprising *H.erectum* and *H.seniawinii* (PP = 0.88, BS = 76%), as shown in Fig. 3.

### ﻿Taxonomy

#### ﻿ Hypericum shunhuangshanense

Taxon classificationPlantaeMalpighiales

J.W. Xiao, W.Q. Qin & W.X. Ouyang sp. nov.

83F3365D-F353-5922-86B1-81496C0C9F8A

urn:lsid:ipni.org:names:77365305-1

Figs 2 , 4

##### Type.

China • Hunan Province, Xinning County, Shaoyang, Shunhuang Mountain Nature Reserve, 1650 m alt., 26.4145, 110.9272, 13 August 2024, *Jiawei Xiao, Wenxiang Ouyang and Zhixin Quan 240813001* (Holotype: Herbarium of Shaoyang University (HSU); Isotype: JIU).

**Figure 4.**
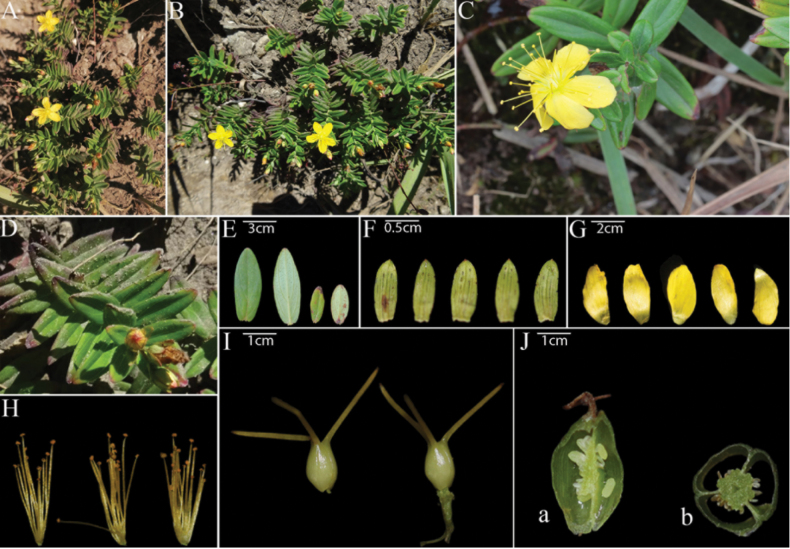
*Hypericumshunhuangshanense*. **A, B.** Habit; **C.** Flower; **D.** Leaves (decussate); **E.** Leaves; **F.** Sepals; **G.** Petals; **H.** Stamens; **I.** Pistils; **J.** Longitudinal section of fruit (a), cross-section of fruit (b).

##### Diagnosis.

*Hypericumshunhuangshanense* is similar to *H.faberi*, *H.seniawinii* and *H.erectum*, but differs from the latter three in the following aspects: its stems are curved or flat and swollen, leaves are decussate, inflorescence is cymose, sepals possess pellucid streaked laminar glands and black marginal glands, and anthers are darker in color.

##### Description.

Perennial herb, 0.1–0.3 m tall. Stems cylindrical, curved or creeping-ascending, usually branched; lower parts reddish-brown, upper parts green. Leaves sessile, 1.7–3.2 cm long, 1.0–1.4 cm wide, oblong to oblong-linear, apex acute to rounded, usually with a small apical point; base cuneate to rounded, margin entire, flat or slightly dark-colored, green above, light green but not glaucous below. Veins: 2 pairs of main lateral veins, each arising from the lower part of the midrib, curving upward in an arc; sparse reticulate veins faintly visible below. Leaf marginal glands black, spaced, rather large. Inflorescence terminal, a capitate cyme with 3–5 flowers; pedicels 0.8–2.8(–5) cm long; bracts small, linear-lanceolate. Flowers 3.0–6.5 cm in diameter. Sepals 5, oblong-lanceolate, 3–5 mm long, 1–2 mm wide, margin entire, midrib distinct, fine veins inconspicuous, laminar glands pellucid, streaks; marginal glands black. Petals golden to lemon yellow, without red tinge, spreading, triangular-obovate, 1.0–1.5 cm long, 0.5–0.7 cm wide, margin entire, laminar glands pellucid, streaks; marginal glands black, few. Stamens in 3 fascicles, 15 stamens per fascicle, longest stamens 1.2–1.5 cm long, equal in length to several petals. Ovary ovoid to pyramidal or subglobose, 1.0–1.5 mm long, 0.5–1.0 mm wide, 3-loculed, with axillar placentation; styles 0.7–1.1 cm long, ca. twice as long as the ovary. Capsule broadly ovoid to narrowly ovoid or subglobose, 6–10 mm long, 4–7 mm wide. Seeds dark reddish-brown, cylindrical, ca. 2 mm long, with a narrow keel-shaped protrusion, surface with shallow linear-reticulate to linear-foveolate ornamentation.

##### Phenology.

Flowering from July to August; fruiting from August to September.

##### Conservation status.

During 2024, we conducted sampling of the *Hypericumshunhuangshanense* population and discovered one additional site near the original discovery location, each harbouring roughly 100 plants. The species primarily inhabits exposed rocky gullies from the mid-section to the summit of the mountain. Further fieldwork is needed to evaluate the exact distribution of the species and it is possible that other populations could be found in similar habitats of the Shunhuang Mountain Nature Reserve. Therefore, we temporarily assign the species to the category DD (Data Deficient) according to the International Union for Conservation of Nature (IUCN 2022).

##### Distribution and ecology.

The species is known to be found in the Shunhuang Mountain Nature Reserve, Xinning County, Shaoyang, Hunan Province, 1650 m alt.

##### Etymology.

The species name “*shunhuangshanense*” refers to the origin of this type, Shunhuang Mountain Nature Reserve, Xinning County, Shaoyang City, Hunan Province.

## Supplementary Material

XML Treatment for Hypericum shunhuangshanense

## ﻿Additional information

### Use of AI

No use of AI was reported.

### Author ORCIDs

Meng-Hua Zhang https://orcid.org/0000-0001-5326-6994

Jia-Wei Xiao https://orcid.org/0000-0001-6541-4413

### Data availability

All of the data that support the findings of this study are available in the main text.
